# Maxillary and Cervical En Bloc Resection With Consideration of the Lymphatic Flow Pathway for Maxillary Gingival Carcinoma: A Case Report

**DOI:** 10.1155/crid/6972361

**Published:** 2026-07-19

**Authors:** Y. Kondo, K. Kawahara, R. Nakamura, Y. Tange, M. Ando

**Affiliations:** ^1^ Department of Oral and Maxillofacial Surgery, Gifu Prefectural Tajimi Hospital, Tajimi, Japan; ^2^ Department of Maxillofacial Surgery, School of Dentistry, Aichi Gakuin University, Nagoya, Japan, agu.ac.jp

**Keywords:** case report, en bloc resection, maxillary cancer

## Abstract

En bloc resection of the primary tumor along with the cervical lymphatic tissue is not commonly performed in patients with maxillary gingival carcinoma because of the physical distance between the primary lesion and cervical metastatic site. However, delayed metastases to lymph nodes outside the standard dissection range, such as the buccinator lymph nodes, may adversely affect the prognosis. We report the case of an 87‐year‐old woman with right maxillary gingival carcinoma with submandibular lymph‐node metastasis in whom en bloc resection of the maxilla and cervical region was performed with inclusion of the buccal space because of concern for possible occult lymphatic spread. Postoperative pathological examination revealed three metastatic submandibular lymph nodes and negative surgical margins. No recurrence was observed for > 5 years after surgery. Buccinator lymph nodes are difficult to identify on preoperative imaging. Therefore, en bloc resection that includes the buccal space may be beneficial in high‐risk cases.

## 1. Introduction

The first‐line treatment for cervical lymph‐node metastasis in oral cancer is surgery. The standard approach involves en bloc resection of the primary tumor and cervical lymphatic tissue, considering the lymphatic drainage route from the primary lesion to the cervical metastasis. However, in maxillary gingival carcinoma, this is technically difficult because of anatomical constraints. Thus, the primary tumor and cervical region are generally resected separately. However, in rare cases, delayed metastasis to lymph nodes outside the typical dissection range, such as the buccinator or lateral retropharyngeal lymph nodes, may contribute to poor prognosis [1–3].

The cervical metastatic pathways in maxillary gingival carcinoma can be broadly divided into two main routes depending on the tumor location [4]. One route passes from the maxillary gingiva through the buccal lymphatics or buccinator lymph nodes to the submandibular lymph nodes. The other extends from the soft palate or nasal floor through the parapharyngeal or retropharyngeal space to the upper internal jugular lymph nodes.

Several reports have described en bloc resection of the maxilla and neck via the parapharyngeal space to prevent delayed metastasis to the lateral retropharyngeal lymph nodes [5–7]. However, to the best of our knowledge, reports on en bloc resection of the maxilla and cervical region including the buccinator lymphatic route are lacking.

Herein, we report a case of maxillary gingival carcinoma with submandibular lymph‐node metastasis in which en bloc resection of the primary lesion and dissected cervical tissue was performed considering the lymphatic drainage route.

## 2. Case Presentation

**Figure 1:**
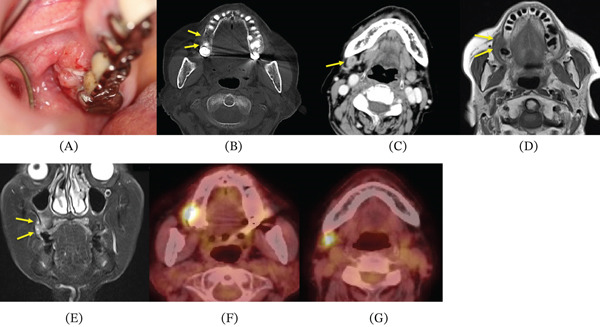
Preoperative imaging. (A) An inward‐growing ulcer measuring 25 × 10 mm is observed, mainly on the buccal gingiva in the right maxillary molar region (Teeth 8‐7‐6). (B) Contrast‐enhanced computed tomography (CT) shows irregular resorption of the buccal alveolar bone in the right maxillary molar region (8‐7‐6). (C) Enlarged right submandibular lymph node (10 mm). Magnetic resonance images show low and high signal intensities on (D) T1‐ and (E) T2‐weighted images, respectively, with evidence of invasion of the buccinator muscle. Positron emission tomography‐CT images show increased fluorodeoxyglucose uptake in the (F) right maxillary molar region and (G) right submandibular lymph node.

An 87‐year‐old woman presented with swelling of the right upper gingiva. The patient had a medical history of hypertension, with no history of smoking or alcohol consumption, was independent in activities of daily living, had good nutritional status, and had an ECOG performance status of one. Intraoral examination revealed a 25 × 10 mm hemorrhagic inward‐growing ulcer in the buccal gingiva of the right maxillary molars (#8–7–6 region) that extended into the buccal mucosa and palatal gingiva (Figure 1A). A firm, enlarged lymph node was palpable in the right submandibular region.

Contrast‐enhanced computed tomography (CT) revealed irregular resorption of the marginal buccal alveolar bone in the #8–7–6 region (Figure 1B) and a 10‐mm diameter, heterogenous right submandibular lymph node (Figure 1C). Magnetic resonance imaging of the right maxillary molar region revealed low and high signal intensities on T1‐weighted and fat‐suppressed T2‐weighted images, respectively, and the lesion extended into the buccinator muscle (Figure 1D,E). Positron emission tomography‐CT showed increased fluorodeoxyglucose uptake in the right maxilla and right submandibular lymph nodes, with maximum standardized uptake values of 10.0 and 7.6, respectively (Figure 1F,G).

Histopathological examination of biopsied tissue confirmed squamous cell carcinoma. The patient was diagnosed with right maxillary gingival carcinoma with submandibular lymph node metastasis (cT2N1M0), and surgery was planned. Because right submandibular lymph‐node metastasis was identified, there was concern regarding possible occult lymphatic spread to the buccal space. Therefore, en bloc resection including the right maxillary tumor, cervical region, and buccal fat tissue was planned.

## 3. Procedure and Clinical Course

**Figure 2:**
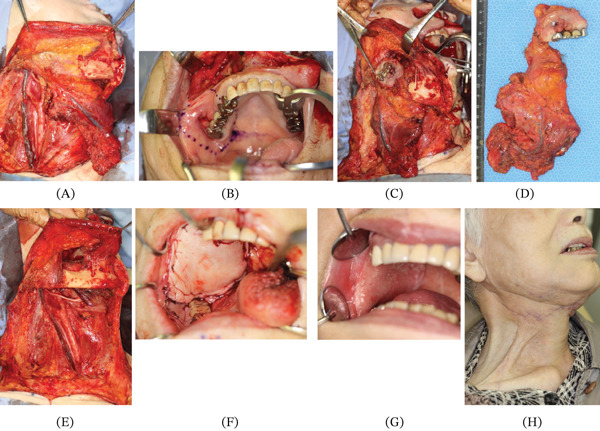
Intraoperative and postoperative photographs. (A) A superior flap has been elevated to maintain continuity between the cervical and buccal‐space tissue. (B) A safe margin has been established around the maxillary tumor. (C) The resected maxillary tumor is connected to the cervical tissue via the buccal space. (D, E) The maxillary tumor, along with buccal‐space and cervical tissues, is resected en bloc. (F) The intraoral defect is reconstructed using a pectoralis major myocutaneous flap. (G) Intraoral photograph and (H) profile at postoperative 6 months.

Cervical dissection was performed first. The cervical incision was then extended, and a midline incision was added in the mental region. Buccal‐space dissection was continuous with the cervical dissection, and deep dissection included the buccinator muscle. The posterior margin was set along the anterior edge of the masseter muscle, the superficial margin followed the superficial layer of the deep cervical fascia, the superior margin was aligned with the buccinator margin at the level of the primary tumor, and the anterior margin was set at the posterior border of the depressor anguli oris muscle. The buccal fat pad along the infrazygomatic crest was dissected to its deep portion and included in the resection (Figure 2A). Next, a partial maxillectomy was performed via a Weber–Ferguson incision. A surgical margin was established around the mucosa surrounding the tumor, and the tumor was resected along with the buccinator muscle. Tooth #4 was extracted, and the bone from Tooth #5 to the maxillary tuberosity was resected (Figure 2B,C). Subsequently, the resected primary lesion and dissected buccal‐space and cervical tissue were removed en bloc (Figure 2D). No communication with the nasal cavity or maxillary sinus was observed at the site of maxillary resection due to the small maxillary sinus volume. Reconstruction of the soft‐tissue defect in the buccal space and oral cavity was achieved using a right pectoralis major myocutaneous flap (Figure 2E,F).

Histopathological examination of the surgical specimen confirmed poorly differentiated squamous cell carcinoma with a depth of invasion of 3 mm, without bone invasion, lymphovascular invasion, or perineural invasion, and with negative resection margins. Three metastatic right submandibular lymph nodes, each < 3 cm in diameter and without extracapsular invasion, were identified and no metastatic lymph nodes were identified in the buccal space (pT1N2bM0, Stage IVA). The operation time was 8 h and 23 min, and the blood loss was 300 g. The patient was discharged from the ICU on postoperative Day 2. The postoperative course was uneventful, with no systemic or flap‐related complications. Oral intake was resumed on postoperative Day 10, and the length of hospital stay was 40 days. This case was not classified as high risk postoperatively, and no adjuvant therapy was administered.

The patient remained recurrence‐free for > 5 years postoperatively, with good control of the primary‐tumor site and cervical region (Figure 2G,H). Although lower lip dysfunction due to facial nerve resection was observed, the maximum mouth opening was approximately 30 mm, and speech, swallowing, and mastication were well preserved. The aesthetic impairment was considered acceptable, and the patient′s quality of life was maintained.

## 4. Discussion

The buccinator lymph nodes are small, inconsistently present facial lymph nodes located between the mandibular and zygomatic lymph nodes [8]. They lie within the buccal fat pad, below the orbit, or anterior to the masseter muscle along the branches of the facial artery and vein. They receive lymphatic drainage from the facial skin and subcutaneous tissues, gingiva, and buccal mucosa and drain into the submandibular lymph nodes. Facial lymph node metastasis in oral cancer is rare, and a previous report found metastases in 0.4% (6/1,406) of oral squamous cell carcinoma cases and 1.4% (6/430) of cases with histologically confirmed lymph node metastasis [9]. However, it is associated with poor prognosis, warranting reconsideration of surgical strategies.

En bloc resection is not commonly performed in patients with maxillary gingival carcinoma because of the considerable anatomical distance between the primary tumor and cervical metastatic sites. However, surgical procedures involving en bloc resection of the primary tumor within the maxilla and cervical region through dissection of the parapharyngeal space have been reported in cases where metastasis to the upper deep cervical lymph nodes via the parapharyngeal lymphatic route was suspected [5–7]. In our patient, invasion of the buccinator muscle by maxillary gingival carcinoma and metastasis to the submandibular lymph nodes were observed, raising the possibility of metastasis via the buccinator lymph nodes [9]. Because buccinator lymph nodes are difficult to identify on preoperative imaging [1], the potential for occult or delayed metastasis was considered. Therefore, prophylactic en bloc resection including the buccal space was selected as part of the initial treatment strategy.

Although reports specifically describing en bloc resection of the maxilla and cervical region including the buccal space are lacking, a comparative study in patients with buccal mucosal carcinoma has been reported. In that study, 53 patients underwent standard resection of the primary lesion with cervical dissection, and 167 patients underwent en bloc resection [10]. The results showed no significant difference in the primary‐tumor control or distant metastasis rates between the two groups; however, the en bloc resection group had significantly better regional lymph node control and 5‐year survival rates. Furthermore, among the 167 patients, 8 were diagnosed as N0 preoperatively but were found to have metastasis to facial lymph nodes. Although the tumor location differs between buccal mucosal cancer and maxillary gingival cancer, some authors have recommended facial lymph node dissection in cases with buccinator muscle invasion because of the potential risk of facial lymph node metastasis [2, 9]. Therefore, en bloc resection may help prevent delayed metastasis in cases without clinically evident micrometastasis.

Nonetheless, en bloc resection for maxillary cancer is not easily adopted in routine practice owing to concerns over postoperative complications associated with the wider resection range. Resection of the buccinator muscle inevitably results in restricted mouth opening and can cause facial motor dysfunction due to damage to the marginal mandibular and buccal branches of the facial nerve. Additionally, because of the communication created between the oral cavity and cervical region, reconstruction of the resulting defect becomes essential. This entails longer operative time, a higher risk of complications, and added burden to the donor site from which the reconstructive flap is harvested. At our institution, we consider en bloc resection for cases of maxillary gingival carcinoma in cases with invasion of the buccinator muscle and confirmed metastasis to submandibular lymph nodes. However, in patients with advanced‐stage lesions, we apply this approach even in the absence of overt metastasis.

This report presents the findings in a single patient, and the efficacy of this surgical method for reducing the incidence of delayed facial lymph‐node metastasis remains unclear. Accumulation of additional cases and continued investigation are necessary.

To achieve more reliable tumor control with the initial‐stage treatment, evaluation of appropriate indications and refining surgical techniques are essential.

## Author Contributions

Y. Kondo: manuscript preparation. K. Kawahara: patient treatment, manuscript editing and review. R. Nakamura: manuscript editing and review. Y. Tange: manuscript editing and review. M. Ando: manuscript editing and review.

## Funding

This work was supported by the Japan Society for the Promotion of Science with grant number JP23K15990.

## Consent

Written patient consent was obtained for the publication of identifiable images and clinical information. Ethics committee approval was not required because this was a single anonymized case report with informed consent.

## Conflicts of Interest

The authors declare no conflicts of interest.

## Data Availability

Data sharing is not applicable to this article as no datasets were generated or analyzed during the current study.
